# Supplementary material for: Content-rich biological network constructed by mining PubMed abstracts
Source: BMC Bioinformatics. 2004 Oct 8;5:147. doi: 10.1186/1471-2105-5-147 (PMC528731; doi:10.1186/1471-2105-5-147)
Supplement: Additional File 5 — The original Chilibot query results of the term "long-term potentiation (LTP)" and 22 other terms, limiting the latest references analyzed to the years 1990, 1995, 2000, and 2004. [file 1471-2105-5-147-S5.bz2 › chilibotAdditionalFile5/ltp2000/html/NMDA_SYNAPTOPHYSIN.html]

 


 **NMDA** and **SYNAPTOPHYSIN** 
  
Found 21 abstracts in PubMed,  **21 abstracts were retrieved and analyzed**.  


---

 Search Google  |
 PDF files only 
|  EDU domain only 

---

**Interactive relationship** (e.g. stimulation, inhibition, etc)

- ErbB 4 colocalizes with PSD 95 and  **NMDA**  receptors at a subset of excitatory synapses apposed to  **synaptophysin**  positive presynaptic terminals.  Ref: 10725395 Proc Natl Acad Sci U S A, 2000
- Fourth, many PSD 95 immunopositive puncta were apposed closely to  **synaptophysin**  positive nerve terminals and overlapped with  **NMDA**  receptor subunits.  Ref: 11027400 J Comp Neurol, 2000

**Parallel relationship** (e.g. studied together, co-existance, homology, etc.)

- We report that long term potentiation was accompanied by increased endogenous glutamate release and increased tyrosine phosphorylation of  **synaptophysin** , but these changes were attenuated when long term potentiation was inhibited by the tyrosine kinase inhibitor tyrphostin AG879 or by the  **NMDA**  antagonist D aminophosphonovalerate.  Ref: 9721920 Neuroreport, 1998
- Studies of gene transcription have provided replicable evidence of decreased expression of mRNAs for  **synaptophysin** , GAP 43, cholecystokinin, and non  **NMDA**  glutamate receptor subunits, particularly in CA 3 4.  Ref: 10071707 Biol Psychiatry, 1999
- Using these antibodies, in conjunction with GluR2 specific and  **synaptophysin**  antibodies, we have directly localized and quantified surface expressed native AMPA and  **NMDA**  receptors on cultured living hippocampal neurons during development.  Ref: 11050112 J Neurosci, 2000
- The distributions of the presynaptic marker  **synaptophysin** , the AMPA type glutamate receptor subunit GluR1, and the putative  **NMDA**  receptor clustering protein PSD 95 were not affected by blockade.  Ref: 9354327 Neuron, 1997
